# Piperine-Loaded In Situ Gel: Formulation, In Vitro Characterization, and Clinical Evaluation against Periodontitis

**DOI:** 10.3390/gels9070577

**Published:** 2023-07-14

**Authors:** Poornima K. Gopalakrishna, Rajamma Abburu Jayaramu, Sateesha Shivally Boregowda, Shruthi Eshwar, Nikhil V. Suresh, Amr Selim Abu Lila, Afrasim Moin, Hadil Faris Alotaibi, Ahmad J. Obaidullah, El-Sayed Khafagy

**Affiliations:** 1 Department of Pharmaceutics, Acharya & BM Reddy College of Pharmacy, Bengaluru 560107, India; poornimakg98@gmail.com; 2 Department of Pharmacognosy, KLE College of Pharmacy, Bengaluru 560010, India; abburjayaramu6@gmail.com; 3 KLE Society’s Institute of Dental Sciences, Bengaluru 560022, India; shruthy2015@gmail.com (S.E.); sureshnikhil596@gmail.com (N.V.S.); 4 Department of Pharmaceutics and Industrial Pharmacy, Faculty of Pharmacy, Zagazig University, Zagazig 44519, Egypt; a.abulila@uoh.edu.sa; 5 Department of Pharmaceutics, College of Pharmacy, University of Hail, Hail 81442, Saudi Arabia; a.moinuddin@uoh.edu.sa; 6 Department of Pharmaceutical Sciences, College of Pharmacy, Princess Nourah Bint AbdulRahman University, Riyadh 11671, Saudi Arabia; hfalotaibi@pnu.edu.sa; 7 Department of Pharmaceutical Chemistry, College of Pharmacy, King Saud University, Riyadh 11451, Saudi Arabia; aobaidullah@ksu.edu.sa; 8 Department of Pharmaceutics, College of Pharmacy, Prince Sattam Bin Abdulaziz University, Al-kharj 11942, Saudi Arabia; e.khafagy@psau.edu.sa; 9 Department of Pharmaceutics and Industrial Pharmacy, Faculty of Pharmacy, Suez Canal University, Ismailia 41522, Egypt

**Keywords:** anti-inflammatory, anti-plaque, in situ gel, periodontitis, piperine

## Abstract

Periodontitis is an inflammatory disorder associated with dysbiosis and characterized by microbiologically related, host-mediated inflammation that leads to the damage of periodontal tissues including gingiva, connective tissues, and alveolar bone. The aim of this study was to develop an in situ gel consisting of piperine. Eight in situ gel formulations were designed by varying the concentration of deacylated gellan gum cross-linked with sodium tripolyphosphate, and poloxamer-407. The prepared gels were evaluated for gelation temperature, gelation time, viscosity, piperine-loading efficiency, and piperine release. Finally, the optimized formula was evaluated for anti-inflammatory effectiveness among human patients during a 14-day follow-up. The optimized in situ gel formulation exhibited a gelation temperature of 35 ± 1 °C, gelling of 36 ± 1 s, excellent syringeability, and piperine loading of 95.3 ± 2.3%. This formulation efficiently sustained in vitro drug release for up to 72 h. In vivo studies revealed an efficient sol-to-gel transformation of optimized in situ gel formulation at physiological conditions, permitting an efficient residence time of the formulation within a periodontitis pocket. Most importantly, a clinical study revealed that treatment with the optimized formulation elicited a significant reduction in the mean plaque score (*p* = 0.001), gingival index (*p* = 0.003), and pocket depth (*p* = 0.002), and exerted a potent anti-inflammatory potential, compared to the control group. Collectively, piperine-loaded in situ gel might represent a viable therapeutic approach for the management of gingival and periodontal diseases.

## 1. Introduction

Periodontitis, also known as gum disease, is a serious gum infection that damages soft tissue surrounding teeth. If left untreated, periodontitis can ruin the bone that supports teeth and may cause other systemic disorders. According to the database reported by Global Burden of Disease (GBD), about 1.1 billion cases of severe periodontitis were reported globally [1]. Periodontitis ranks 77th among the most relevant human conditions which results in disability. Nevertheless, gum problems are resolvable if timely intervention is provided [2].

Generally, periodontitis is associated with dysbiosis [3] and characterized by microbially associated, host-mediated inflammation that leads to the damage of periodontal tissues including gingiva, connective tissues, and alveolar bone [4]. Ultimately, this activates host-derived proteinases, facilitating the apical migration of the junctional epithelium, the loss of marginal periodontal ligament fibers, and the anterior spread of the bacterial biofilm along the root surface [5]. Some techniques, such as guided bone regeneration (GBR), have been adopted for periodontal regeneration via creating a favorable environment for bone regeneration by preventing the migration of soft tissue cells into the defect and allowing space for bone-forming cells to populate the area [6,7]. For instance, periosteum-inspired composite membranes based on sodium alginate-hydroxyapatite nanoparticles [8] and chitosan/bioactive glass nanoparticle composite membranes [9] have been investigated for their potential use in periodontal regeneration. However, GBR represents a complex proliferation process in which different types of cells including osteoblasts, osteocytes, osteoclasts, and bone-lining cells should orchestrate to regulate bone formation.

Commonly, conventional periodontal therapy involves oral hygiene instruction, scaling with root planning, and surgical intervention [10]. Nevertheless, periodontal patients who do not respond to conventional mechanical therapy, or are suffering from acute periodontal infections with systemic manifestations are usually advised to be treated with systemic antibiotics. Various antibiotics, such as tetracyclines [11], moxifloxacin [12], doxycycline [13], etc., have been prescribed for the systemic treatment of periodontitis. Nevertheless, the usage of these drugs was associated with certain drawbacks such as the inability of systemic drugs to attain adequate gingival crevicular fluid concentration [14], the development of drug resistance [15], an increased risk of drug-related adverse effects [16], and questionable patient compliance [17].

Apart from the systemic administration of drugs, localized drug delivery in the form of fibres, strips, films, and micro-particulate systems has been widely investigated for the management of periodontitis [18,19,20]. Nevertheless, they show limited success in the treatment of periodontitis because of poor patient compliance, poor adhesion properties, and limited retention time [21]. Most importantly, the difficulty in removing such systems after the completion of treatment without causing inflammation and gingival redness adversely limited their widespread usage for the treatment of periodontitis.

Because of their unique biomimetic properties, hydrogels are employed as biomaterials in tissue engineering and regenerative medicine to enhance cell adhesion and promote tissue regeneration. Recently, the use of natural-origin materials, such as collagen, gelatin, polysccahrides, etc., for the formulation of hydrogels has attracted great attention due to their ability to mimic the native tissues’ extracellular matrix and their biocompatibility [22]. In situ gelling systems (ISGs) are polymeric viscous hydrogels that, when applied to the human body, undergo a sol-to-gel transition in response to different stimuli such as ionic strength, temperature, or pH [23]. Recently, ISGs have emerged as a promising local drug delivery system for the management of periodontitis because of their ability to attain high drug levels in the gingival crevicular fluid for long periods of time, promoting the achievement of the desired therapeutic effect [24]. In addition, in situ gels are easy to administer and undergo rapid elimination from the cavity through normal catabolic pathways after the complete delivery of the drug [25]. In situ gel can be categorized into three classes according to its phase change performance: pH-sensitive [26], temperature-sensitive [26], and ionic-strength-sensitive [27]. Among them, ion-sensitive materials such as sodium alginate is the most commonly used. However, in recent years, deacetylase gellan gum has gained popularity [27]. Deacetylase gellan gum is an extracellular polysaccharide, produced from *Pseudomonas elodea* bacteria. It exhibits very high gel strength, adjustable gel elasticity, high gel transparency, and good compatibility. Because of its distinct characteristics, it has been employed for oral [28], ocular [29], and nasal administration systems [30].

Piperine is the primary alkaloid isolated from long pepper (*Piper longum*) and black pepper (*Piper nigrum*) [31]. Piperine has versatile biological activities including analgesic, anti-inflammatory, antitumor, anticonvulsant, and neuroprotective activities [32,33,34]. In addition, piperine has been screened for its antimicrobial activity against various microbes. It has been verified to be effective against *Escherichia coli*, *Staphylococcus aureus*, *Pseudomonas aeruginosa*, and *Bacillus subtilis* [35]. Furthermore, piperine has been reported to exert significant protective effects on inflammation, bone and collagen fiber degeneration, and alveolar bone loss in a rat periodontitis model [36].

The aim of this study, therefore, was to develop and optimize piperine-loaded in situ gel and clinically evaluate its potential in the treatment of periodontitis. In situ gel formulations were fabricated using the biodegradable biocompatible polymer, deacylated gellan gum, cross-linked with sodium tripolyphosphate, and poloxamer-407. The formulated gels were physico-chemically characterized, and the optimized formula was eventually evaluated for anti-inflammatory effectiveness among human patients suffering from periodontitis.

## 2. Results and Discussion

### 2.1. Pre-Formulation Studies

**Figure 1:**
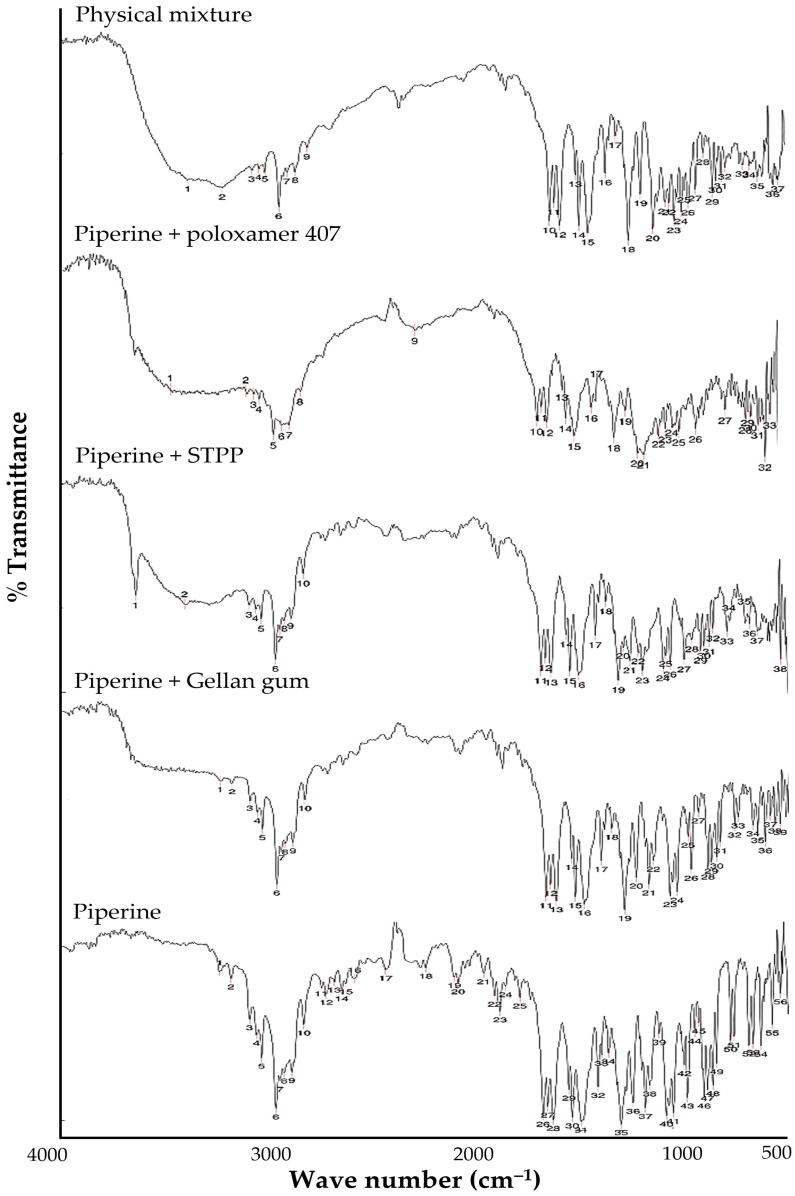
FTIR spectra of pure piperine and different formulation excipients.

FTIR spectral analysis was carried out to study piperine compatibility with other formulation excipients. The FTIR spectra of pure piperine, gellan gum, STPP, poloxamer 407, and the physical mixture of the drug with excipients are depicted in Figure 1. The characteristic spectral peaks of N-H stretching at 3214.75 cm^−1^, C=C aromatic stretching at 1489 cm^−1^, and aromatic -C-H stretching at 2937.3 cm^−1^ were observed in the spectrum of pure piperine [37]. In the spectrum of the physical mixture of piperine with various excipients, no remarkable changes were observed in the absorption peaks at 3213.7 cm^−1^, 1491 cm^−1^, and 2940 cm^−1^, corresponding to N-H stretching, C=C aromatic stretching, and aromatic -C-H stretching, compared to those of pure piperine. From the FTIR interpretation, it was evident that there was no interaction between the drug and various formulation excipients. Therefore, this study claimed that piperine was compatible with various formulation excipients.

### 2.2. In Situ Gel Formulation

Gellan gum is an ion-sensitive natural polysaccharide, which consists of double helical segments [38]. These segments can cross-link in the presence of monovalent or divalent cations to form biocompatible hydrogels [39]. However, the ion content of gingival crevicular fluid (GCF) in the periodontal pocket was found to be insufficient to initiate the gelation of the gellan gum. Accordingly, for the formulation of gellan-gum-based in situ gel, sodium tripolyphosphate (STPP) was adopted as a cross-linking agent that helps the formation of a stiff gel at the body temperature. The cross-linking process involves coacervation complexation through electrostatic attraction between negatively charged gellan gum with the positively charged STPP [40,41]. Therefore, various trials were conducted by varying the concentration of gellan gum (from 0.5 to 1% *w/v*) and STPP concentration (from 0.2 to 1% *w/v*), and the consistency of the prepared in situ gel formulations was visually inspected (Table 1). As summarized in Table 1, both gellan gum and STPP concentrations exerted a significant impact on the sol–gel transition of the formed in situ gel formulations. Gellan gum at concentrations of 0.5 to 0.75% *w/v* and STPP at concentrations of 0.2 to 0.4% *w/v* succeeded to produce formulations with a gel-like consistency at 35 ± 2 °C. On the other hand, regardless of STPP concentrations, formulations with gellan gum, at a concentration of 1% *w/v*, were found to be gel even at room temperature (non-physiological condition); accordingly, they were excluded from further investigations. 

It is worth noting that physically cross-linked gellan gum hydrogels might lose their stability under physiological conditions, limiting their in vivo use. Consequently, in order to enhance the mechanical strength of the gels, the thermo-responsive polymer, poloxamer 407, was incorporated as a co-polymer during the formulation of in situ gels. Poloxamer 407 was chosen because it has unique reversible thermo-gelling properties [42]. In addition, it has an excellent safety profile and good mucoadhesive properties [43]. Furthermore, gellan gum along with poloxamer 407 can transform into stiff gel by the combined effect of body temperature and the ions available in the periodontal fluid. Accordingly, a total of eight formulations were prepared (Table 2) by incorporating poloxamer 407, at concentrations of 10 and 12% *w/v*, into STPP-cross-linked gellan gum, and, eventually, physico-chemically evaluated for the selection of an optimized formula.

### 2.3. Evaluation of In Situ Gel

#### 2.3.1. Gelation Temperature and Gelation Time

**Figure 2:**
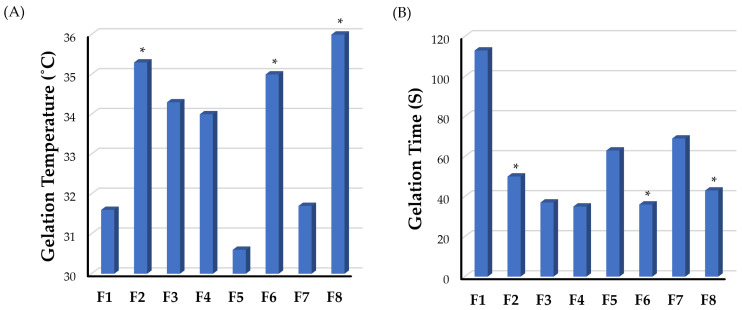
Impact of formulation components on (**A**) gelation temperature and (**B**) gelation time of various piperine-loaded in situ gel formulations. Comparisons were conducted among different formulations having the same gellan gum and STPP concentration. * *p* < 0.05.

The gelation temperature of in situ gelling systems is one of the critical characteristics that dictate their in vivo applicability. It is ideal for in situ gelling systems to exist in a sol state at room temperature to permit easy drug administration, and then be rapidly converted into a gel state once in the periodontal cavity. Furthermore, these gels should not dissolve but rather remain in a gel state for an extended period of time [44]. Generally, the gelation temperatures of in situ gel formulations have been deemed appropriate if they are within the range of body temperature [45]. The gelation temperatures of different gellan-gum-based in situ gel formulations are represented in Figure 2A and summarized in Table 2. As depicted in Table 2, all formulations (F1–F8) showed an acceptable gelation temperature, which is higher than room temperature and lower than body temperature, ranging from 30.6 ± 0.6 to 37.0 ± 1.0 °C. In addition, it was evident that the gelation temperature of formulations increased with an increase in the concentration of the thermosensitive co-polymer poloxamer 407. The gelation temperature of F1 (31.6 ± 0.6 °C), prepared with a poloxamer 407 concentration of 10% *w/v*, was remarkably lower than that prepared with a poloxamer 407 concentration of 12% *w/v* (F2; 35.3 ± 0.6 °C) (*p* < 0.05). Similarly, formulations F6 and F8, containing poloxamer 407 (12% *w/v*), showed excellent gelation compared to F5 and F7, containing poloxamer 407 (10% *w/v*), respectively, due to increasing the concentration of poloxamer 407 for the same gellan gum concentration. However, the gelation temperature of F3 and F4 formulations was not influenced by poloxamer 407 concentration. Similar results were stated by Rencber et al. [46] who verified the positive effect of increasing the concentrations of the thermosensitive polymer, poloxamer 407, on the gelation temperature of gellan-gum-poloxamer-based dexamethasone mucoadhesive in situ gel.

Gelation time is another crucial characteristic of in situ gel systems. Generally, a shorter gelation time would be advantageous in minimizing the time necessary for the implanted dosage to turn into a viscous gel. Therefore, this would reduce the chance of the implanted formulation to be rapidly diluted by the gingival crevicular fluid and being lost with salivary secretions. The gelation time of the prepared in situ gel formulations was represented in Figure 2B and Table 2. As illustrated in Table 2, gelation times of various formulations fluctuate between 35.0 ± 1.0 s (F4) to 113.0 ± 2.1 s (F1). In addition, it was inferred that gelation times were dependent on the poloxamer 407 concentration. At the same gellan gum concentrations, increasing the poloxamer 407 concentration from 10 to 12% *w/v* resulted in an obvious reduction of gelation time of different formulations. The gelation time of F2, prepared with 12% poloxamer 407, was 50.0 ± 1.5 s, which was significantly lower than that of F1 (113.0 ± 2.1 s) prepared with 10% poloxamer 407 (*p* < 0.05). Similarly, formulations F4, F6, and F8, containing poloxamer 407 (12% *w/v*), showed remarkably lower gelation time compared to F3, F5, and F7, respectively, containing poloxamer 407 (10% *w/v*), due to increasing the concentration of poloxamer 407 for the same gellan gum concentration. Similar findings were reported for levofloxacin-poloxamer 407 gels, where gelation time were dependent on poloxamer concentrations [47].

#### 2.3.2. Viscosity Measurement

The quantity of the gel that can be introduced into the periodontal pockets is extremely low due to small crevices. Consequently, the viscosity of the in situ gel must be low at the time of application to the periodontal pockets to ease formulation application, but high thereafter in order for the drug to remain for a sufficient amount of time in the disease site [26]. The viscosities of different formulations at both room temperature and at elevated temperatures were measured and compared. As anticipated, due to its thermo-responsiveness, the viscosities of all formulations at room temperature were significantly lower than those at elevated temperatures (37 ± 0.5 °C) (Table 2). The viscosities of test formulations at room temperature (in the sol form) were within the range of 58.3 ± 1.7 cp (F6) to 59.6 ± 1.2 cp (F7), while, at 37 ± 0.5 °C, the viscosities of test formulations fluctuated between 243.0 ± 19.8 cp (F2) and 290.2 ± 22.6 cp (F3). Such significant increase in the formulations’ viscosities might be ascribed to the transition of in situ gel systems from the sol state at room temperature to the gel state at 37 ± 0.5 °C, which is higher than the gelation temperature of all test formulations. These results confirm that the formulation can be easily administered into the periodontal pockets where it will be rapidly converted into the gel state at the physiological temperature.

#### 2.3.3. pH Measurement

In the oral cavity, the pH is maintained near neutrality (6.7–7.3) by saliva. Acidic or alkaline formulations may cause irritation to the buccal mucosa; consequently, the pH is considered as a critical parameter in the formulation of buccal dosage forms [4,26]. The pH of all in situ gel formulations containing piperine was found to fluctuate between 7.0 (F5) and 7.8 (F4) (Table 2). The reason behind the relatively basic pH of the gel is due to the cross-linking of the gellan gum with STPP. STPP is a basic component, which imparts an alkaline pH to the formulation. As depicted in Table 2, formulations cross-linked with 0.4% *w/v* STPP showed relatively higher pH values compared to those cross-linked with 0.2% *w/v* STPP. Nevertheless, since the pH of periodontal fluid is around 7.2 to 7.8, all formulations are deemed to be biologically compatible and would avoid irritation upon application in periodontal cavities.

#### 2.3.4. Syringeability Study

A syringeability test was performed to ensure that the produced formulations had a good solution flow nature and could be utilized to deliver the formulation to the periodontal pocket. Syringeablity mainly depends on the concentration of the polymer and viscosity. Herein, all the developed formulations showed good syringeability as manifested by an easy and continuous flow through a 24-gauge needle at room temperature (Table 2). These results verify the easy application of the prepared in situ gel formulation to the disease site in vivo.

#### 2.3.5. Drug Content Percentage

The drug content is one of the important parameters, which directly influences the drug release property. The drug content of the formulations was found to be varied from 57.5 ± 1.9% (F5) to 95.3 ± 2.3% (F6) (Table 2). Such variation in drug content percentage might be ascribed to the degree of cross-linking of gellan gum with STPP. At higher STPP concentrations, extensive cross-linked gellan gum would occur, resulting in a significant reduction in the pore size of the gel, which, in turn, would favor piperine retention within the gel structure.

### 2.4. In Vitro Drug Release

**Figure 3:**
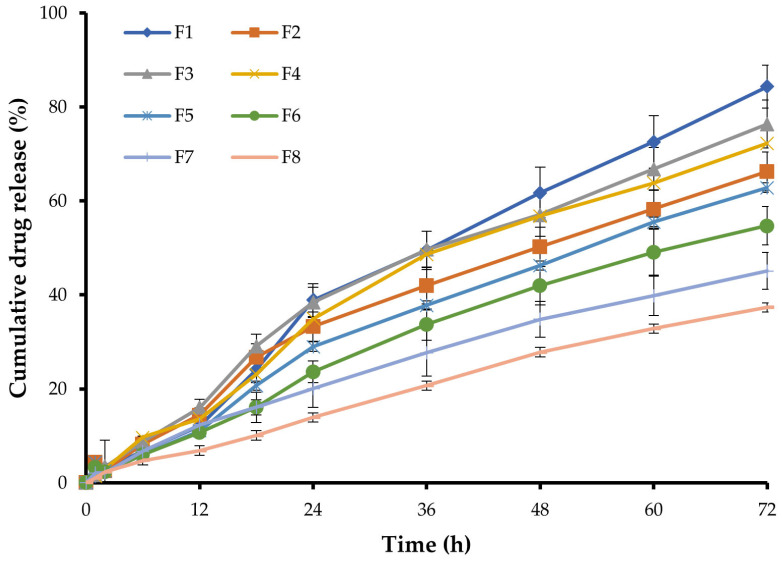
In vitro release profiles of piperine from various in situ gel formulations.

An in vitro release study is a pre-assessment study to understand the release pattern of the drug at body conditions. The in vitro release profiles of piperine from different in situ gel formulations are represented in Figure 3. As depicted in Figure 3, the release of piperine was in the range of 37.3 ± 2.9% (F8) to 84.3 ± 4.6% (F1) over 72 h. Variations in the percentage cumulative drug release at 72 h was directly related to formulation composition. Increasing gellan gum concentration from 0.5% *w/v* to 0.75% *w/v* significantly retarded drug release from the gel matrix. The percentage cumulative drug release from F1, prepared with 0.5% *w/v* gellan gum, was 84.3 ± 4.6%, which was significantly higher than that from F5 (62.9 ± 3.7%), prepared with 0.75% *w/v* gellan gum. In the same context, increasing poloxamer 407 concentration from 10 to 12% *w/v* remarkably slowed piperine release from the gel matrix. The percentage of the drug released from F2, prepared with 12% *w/v* poloxamer 407, was 66.2 ± 4.1%, which was significantly lower than that from F1 (84.3 ± 4.6%), prepared with 10% *w/v* poloxamer 407. Poloxamer 407 was reported to act as a release barrier within the gel matrix via reducing the number and dimension of water channels and increasing the number and size of micelles within the gel structure [24]. This clearly explains the retarding effect of poloxamer 407 on piperine release from various in situ gel formulations.

The in vitro release data were kinetically analyzed according to the zero-order, first-order, and Higuchi model. The relatively high correlation coefficient (R^2^) values obtained with the zero-order model suggested the release of fixed drug amounts at fixed time intervals. Accordingly, it can be anticipated that, among various formulations, F6 would sustain piperine release under in vivo conditions for up to 7 days, while other formulations, such as F1, F3, and F4, would be exhausted early and may not sustain drug release for 7 days as desired.

Finally, based on various in situ gel characteristics, formulation F6, which shows reasonable physico-chemical characteristics such as good gelation temperature (35.0 ± 1.0 °C), short gelling time (36.0 ± 1.0 s), appropriate pH (7.4 ± 0.3), high drug content (95.3 ± 2.3%) along with efficient sustained drug release, was selected as an optimized formula to be used in further investigations.

### 2.5. DSC Studies

**Figure 4:**
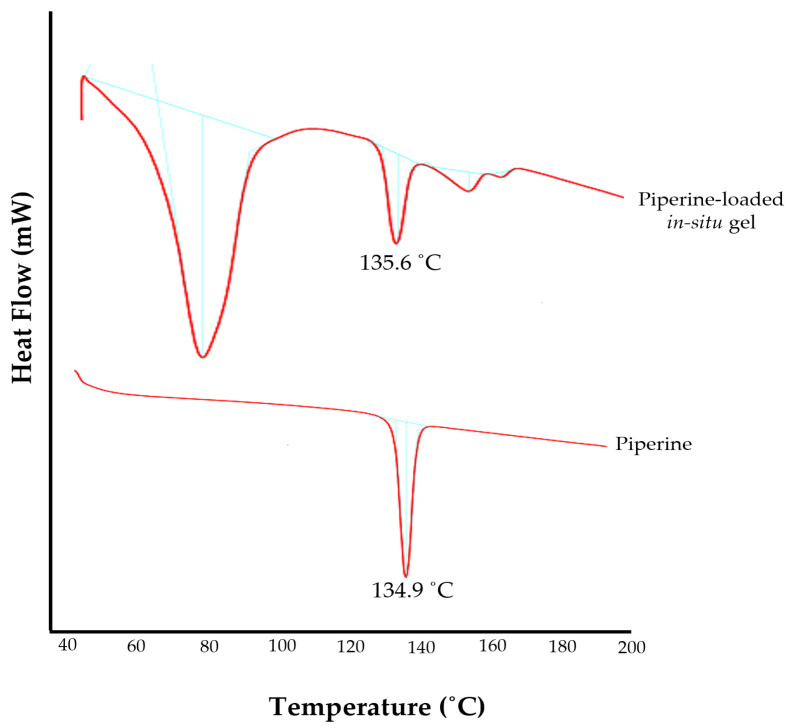
DSC thermograms of pure piperine and optimized piperine-loaded in situ gel formulation.

DSC is an important analytical tool used to study the thermal effect, physical transitions, solid–solid transitions, and compatibility of the drug with formulation ingredients. The DSC thermogram of the pure drug showed an endothermic peak at 134.9 °C, corresponding to its melting point (Figure 4) [48]. The endothermic peak of the piperine in the optimized in situ gel formulation (F6) was slightly shifted to 135.6 °C (Figure 4). This result infers that piperine is compatible with the excipients of the formulation, and no significant interaction or modification in drug properties occurred upon its incorporation in the formulation.

### 2.6. Clinical Evaluation of Piperine Gel in Human Patients

Dental deposits are the major concern for soft tissue inflammation. Recently, herbal products used in traditional medicine have gained popularity in the field of dental disease prevention. They can provide safe and long-term solutions for maintaining good dental health. Hence, in this study, the anti-plaque and anti-gingival effectiveness of piperine-loaded in situ gel was assessed in vivo. A non-randomized controlled clinical trial was conducted among 30 subjects, divided into two groups: Group 1 receiving the optimized piperine-loaded in situ gel formulation (F6) following oral prophylaxis, and Group 2 treated with oral prophylaxis alone. The demographic characters of the study participants were presented in Table 3.

**Figure 5:**
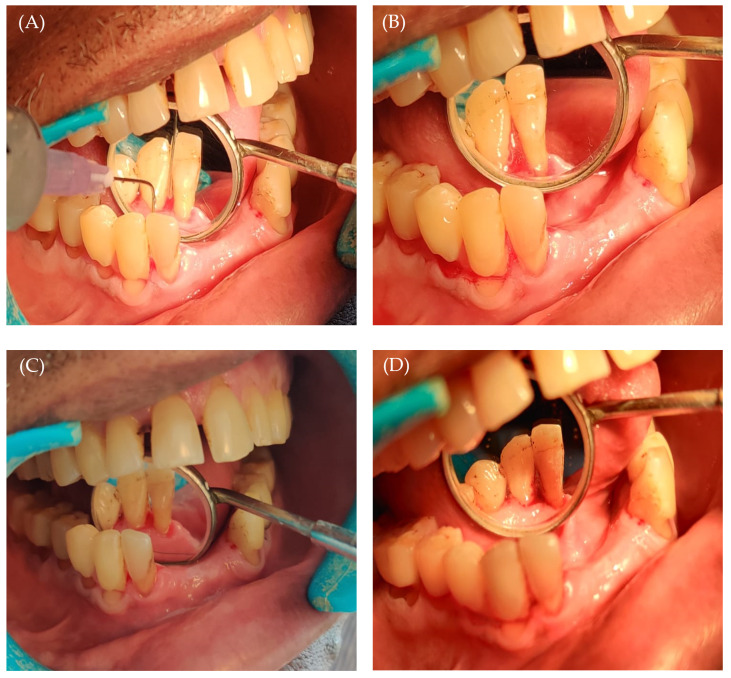
(**A**) Application of piperine in situ gel; (**B**) gelation of piperine in situ gel inside the socket; (**C**) intra-oral image post oral prophylaxis; and (**D**) follow-up after 14 days.

All the subjects were evaluated for plaque score, gingival index, and pocket depth at baseline and 14-day follow-up. At the baseline, there was no significant difference between the two groups in the mean plaque score (Group 1: 1.67 ± 0.49 vs. Group 2: 1.89 ± 0.31; *p* = 0.140), gingival index (Group 1: 1.70 ± 0.45 vs. Group 2: 1.99 ± 0.32; *p* = 0.053), and pocket depth (Group 1: 5.47 ± 0.52 vs. Group 2: 5.53 ± 0.52; *p* = 0.483) (Table 4). On the other hand, at the 14-day follow-up, both groups exhibited a reduction in mean plaque score, gingival index, and pocket depth, compared to baseline values (Figure 5). Interestingly, there was a significant reduction in the clinical parameters on the 14-day follow-up in Group 1 compared to Group 2. As depicted in Table 4, at the 14-day follow-up, the plaque score showed a significant reduction in Group 1 compared to Group 2 (*p* = 0.0001). Similarly, the gingival index was significantly reduced in Group 1 (*p* = 0.0003). Most importantly, the pocket depth in Group 1 was reduced to 3.27 ± 0.59 mm at the follow-up, from 5.47 ± 0.52 mm at baseline, which was statistically lower than that in Group 2 (*p* = 0.0002). These results might be correlated with the in vitro release results, which advocate the sustained release of piperine from in situ gel formulation.

Piperine has a well-documented anti-inflammatory effect. However, few researchers have studied its potential use in periodontitis disease [49]. In this study, we confirmed the efficacy of piperine-loaded in situ gel in fighting plaque and reducing gingival inflammation and periodontal pocket depth. Such effects could be attributed to the anti-inflammatory and anti-bacterial activities of piperine. Piperine has been reported to inhibit nitric oxide and tumor necrosis factor-alpha production, both of which are known to play roles in the pathophysiology of inflammation in periodontal disease [50]. In addition, piperine has been proven to have a substantial anti-biofilm impact on *Streptococcus mutans* [51]. Nevertheless, it is worth noting that there are few limitations in our clinical study. The short study duration, along with the limited number of study participants might hinder the generalization of the study results to the whole population. However, within the limitations of the study, piperine-loaded in situ gels proved efficient anti-plaque, anti-gingival, and anti-inflammatory activities in patients with periodontitis (Figure 5 and Table 4). Further studies are required to assess the long-term benefits of our in situ gel formulation in treating periodontitis.

## 3. Conclusions

In this study, in situ gel formulations containing piperine was developed with a combination of gellan gum and poloxamer 407 and evaluated as a potential drug delivery system for the treatment of periodontitis. The optimized formulation exhibited an optimal phase transition temperature, short gelling time, and appropriate viscosity at room temperature, which enable the easy application of the formulation. In addition, under physiological conditions, the optimized formulation was efficiently transformed into a hard gel that could sustain drug release for up to 72 h. Most importantly, a non-randomized controlled clinical trial revealed a significant decline in mean plaque score, gingival index, and pocket depth among the test group from baseline to 14 days, whereas this was not significant in the control group. Collectively, a combination of polymers having different mechanisms of in situ gelation might represent a viable delivery vehicle for the application of drugs used for the management of periodontitis.

## 4. Materials and Methods

### 4.1. Materials

Piperine and sodium tripolyphosphate were procured from Karnataka Fine Chemicals (Bangalore, India). Poloxamer-407 was procured from Sigma Aldrich (St. Louis, CA, USA). Gellan gum (molecular weight 500 kDa, 95% deacylation degree) was a gift from Sisco Research Laboratories (Mumbai, India). All other reagents were of analytical grade.

### 4.2. Drug-Excipient Compatibility by Fourier-Transform Infrared (FTIR) Spectroscopy

In order to check the physico-chemical compatibility between piperine and different formulation excipients, FTIR analysis was conducted. Briefly, piperine was mixed with formulation excipients at a ratio of 1:1 and examined by BRUKER FTIR spectrometer (TENSOR; Markham, ON, Canada). The spectra of the samples were recorded and analyzed at the wavenumber range of 4000 cm^−1^ to 400 cm^−1^.

### 4.3. In Situ Gel Formulation

Gellan gum (0.5–1% *w*/*v*) was dispersed in deionized water and then heated to 60–80 °C with agitation at 150 rpm for 2 h until total dissolution [52]. Sodium tripolyphosphate (STPP; 0.2–1% *w*/*v*) was added as a cross-linking agent [53] to the gellan gum solution and the dispersion was maintained at 65 °C with agitation at 150 rpm for 1 h. The obtained solution was then brought to 8 ± 2 °C on ice bath. Polaxomer-407 (10–12% *w*/*v*) was added as co-polymer [54] to the above solution and stirred at 100 rpm for 1 h until solution becomes clear. Hydro-alcoholic solution of piperine was finally added to the above dispersion with stirring for 30 min to obtain the overall concentration of piperine of 0.4% *w*/*v*. The piperine-dispersed in situ gel was then stored in refrigerator between 2 to 8 °C for further studies (Table 1).

### 4.4. In Vitro Evaluation of Piperine-Loaded In Situ Gel

#### 4.4.1. Gelation Temperature

Gelation temperature is the temperature at which the preparation becomes thick, stiff, and resistant to flow. The gelation temperature of the formulation was determined by test tube inversion method [55]. Briefly, 5 mL in situ gel was placed in a test tube, containing 0.5 mL of 0.1% *w*/*v* NaCl, and incubated in a temperature-controlled water bath maintained at 15 ± 2 °C. The temperature of the water bath was then gradually raised to 40 °C, with an increment of 2 degrees every 5 min. At each set point, the test tube was inverted at 90°, and the temperature at which no flow was observed upon inversion was recorded as a gelation temperature.

#### 4.4.2. Gelling Time

Gelling time of different formulations was measured by test tube inversion method [43]. Briefly, 5 mL of each formulation was added into a glass test tube containing 0.5 mL of 0.1% *w*/*v* NaCl. The test tube was incubated in a temperature-controlled water bath set at 35 ± 2 °C. The test tubes were periodically inverted at 90° and the time taken for the liquid to transform to gel (no flow) was recorded as gelling time in sec [56].

#### 4.4.3. Gel pH

The pH of developed in situ gel formulation was estimated using calibrated digital pH meter (Symbiont Life Sciences, Chennai, India). The pH meter was calibrated before its use with standard buffer solutions. The pH was measured by dipping the electrode sufficiently into the formulation at room temperature. The evaluation was carried out in triplicate and results are recorded as average of three trials.

#### 4.4.4. Gel Viscosity

Viscosity of in situ gel formulations was measured using Brookfiled DVE viscometers (Brookfield Engineering, Middleboro, MA, USA). Various trials were conducted to choose the appropriate spindle for analyzing the samples. Based on the % Torque value, spindle 61 was selected to measure the viscosity of the liquid and spindle No. 62 to measure gel viscosity. The viscosity of the liquid was measured at 25 ± 2 °C, while the gel was evaluated at 37 ± 0.5 °C.

#### 4.4.5. Syringeability

A constant volume of 1 mL of test formulation, maintained at 25 ± 2 °C, was filled into 1 mL syringe attached with 24-gauge needle. The solution which easily passed from syringe was termed as “pass” and those which had difficulty passing through were termed as “fail” [57].

#### 4.4.6. Drug Content of the Gel

The piperine content in the in situ gel formulation was determined by UV spectroscopy (Agilent Technologies, Santa Clara, CA, USA). Briefly, 1 mL of the gel formulation was diluted to 10 mL with ethanol and sonicated using bath sonicator until a clear solution is obtained. Solution was filtered and piperine content in the in situ gel was determined at 343 nm using ethanol as a blank [58].

### 4.5. Differential Scanning Calorimetric (DSC) Studies

Thermoanalytical curves of piperine and the in situ gel of piperine were obtained using a METTLER TO-143 LEDO differential scanning calorimeter (Columbus, OH, USA). Samples (4–6 mg) were placed in hermetically sealed aluminum cells. The samples were heated and scanned in the range of 20 to 200 °C at a heating rate of 10 °C min^−1^ under a nitrogen atmosphere with a flow rate of 80 mL min^−1^. Heat flow versus temperature plots were developed to obtain the spectra [59].

### 4.6. Drug Release Studies

In vitro drug release profiles of in situ gel were determined using 250 mL capacity Franz diffusion cell. The dialysis membrane with an aperture size of 0.45 µ was interfaced between the donor and receptor cells. Then, 1 mL of in situ gel was placed in the donor cell. The receptor compartment was filled with 250 mL of phosphate buffer solution (PBS, pH 7.4) kept at 37 ± 0.5 °C and constantly stirred at 25 rpm. At scheduled time points (1, 2, 6, 12, 18, 24, 36, 48, 60, and 72 h), 3 mL samples were withdrawn and replenished with the same volume of pre-warmed PBS to maintain the sink condition. Drug concentration in collected samples was quantified spectrophotometrically at λ_max_ 343 nm.

### 4.7. Randomized Clinical Trial

#### 4.7.1. Ethical Clearance

The study proposal was submitted for approval and clearance was obtained from the Institutional Human Ethical Committee (No. KIDS/IEC/May2023/27), K.L.E Society’s Institute of Dental Sciences, Bangalore, India. Written informed consent was obtained from each of the participating subject.

#### 4.7.2. Study Design and Study Setting

A non-randomized controlled clinical trial was conducted among 30 subjects in the age range of 20–50 years. The study was carried out on subjects aged 20 to 50 years with moderate to severe chronic periodontitis (AAP classification, 1999) and at least three nonadjacent interproximal sites with a probing pocket depth of 6 mm, and no contraindication to periodontal treatment were included in the study. Patients had to have not undergone any periodontal or antibiotic therapy in the previous 6 months and be co-operative and dedicated to maintaining good dental hygiene. Patients who smoked, used tobacco, or drank alcohol, had a systemic ailment, were pregnant or breastfeeding, had used an antimicrobial mouthwash in the previous two months, or needed periodontal surgery were excluded from the trial.

#### 4.7.3. Study Procedure

A total of 30 patients, divided into two groups, were subjected to this study. Prior to starting the investigation, demographic characters (Table 3) were estimated through interview method. The clinical parameters including plaque index, gingival index, and periodontal pocket depth were evaluated in every individual [60]. Group 1 (test group) included 15 participants who received SRP (scaling and root planning) with piperine gel intervention. Group 2 (control group) included 15 participants who only received SRP. In the intervention group (Group 1), after finishing scaling and root planning, the in situ gel was applied into the pocket depth. For in situ gel application, a syringe with a blunt needle was used. The gel was gently released while the needle was pushed coronally from the bottom of the pocket. In the control group, SRP alone was performed, and oral hygiene instructions were given to subjects belonging to both the groups. Patients were recalled on 14th day. All clinical parameters were recorded during the recall visit.

### 4.8. Statistical Analysis

Data analysis was achieved using the SPSS 26.0 (SPSS Inc., Chicago, IL, USA). Statistical analysis was carried out by Student’s *t*-test, paired *t*-test, and Wilcoxin test. A *p* value < 0.05 is considered significant.

## Figures and Tables

**Table 1:** Polymeric combination of gellan gum and STPP and their consistency.

Trials	Gellan Gum (% *w/v*)	STPP (% *w/v*)	Consistency at 25 ± 2 °C	Consistency at 35 ± 2 °C
1	0.5	0.2	Liquid	Gel
2	0.5	0.4	Liquid	Gel
3	0.5	0.6	Liquid	Liquid
4	0.5	0.8	Liquid	Liquid
5	0.5	1.0	Liquid	Liquid
6	0.75	0.2	Liquid	Gel
7	0.75	0.4	Liquid	Gel
8	0.75	0.6	Liquid	Viscous solution
9	0.75	0.8	Liquid	Viscous solution
10	0.75	1.0	Liquid	Liquid
11	1.0	0.2	Gel	Gel
12	1.0	0.4	Gel	Gel
13	1.0	0.6	Gel	Gel
14	1.0	0.8	Gel	Gel
15	1.0	1.0	Gel	Gel

**Table 2:** Composition and physico-chemical characteristics of piperine-loaded in situ gel formulations.

Formula	Gellan Gum	STPP	Poloxamer 407	Gelation Temperature (°C)	Gelation Time (s)	pH	Syringeability	Drug Content (%)	Viscosity at 25 °C (cps)	Viscosity at 37 °C (cps)
F1	0.5	0.2	10	31.6 ± 0.6	113.0 ± 2.1	7.2 ± 0.1	Pass	62.5 ± 1.7	58.7 ± 1.9	287.9 ± 21.3
F2	0.5	0.2	12	35.3 ± 0.6	50.0 ± 1.5	7.0 ± 0.3	Pass	87.6 ± 2.9	58.8 ± 1.4	243.0 ± 19.8
F3	0.5	0.4	10	34.3 ± 1.2	37.0 ± 1.5	7.6 ± 0.4	Pass	92.2 ± 2.1	59.4 ± 2.1	290.2 ± 22.6
F4	0.5	0.4	12	34.0 ± 1.0	35.0 ± 1.0	7.8 ± 0.1	Pass	82.5 ± 3.6	59.3 ± 1.2	287.0 ± 17.8
F5	0.75	0.2	10	30.6 ± 0.6	63.0 ± 1.2	7.0 ± 0.1	Pass	57.5 ± 1.9	59.2 ± 1.6	257.0 ± 13.4
F6	0.75	0.2	12	35.0 ± 1.0	36.0 ± 1.0	7.4 ± 0.3	Pass	95.3 ± 2.3	58.3 ± 1.7	264.0 ± 16.8
F7	0.75	0.4	10	31.7 ± 0.6	69.0 ± 2.5	7.5 ± 0.2	Pass	95.1 ± 1.8	59.6 ± 1.2	280.0 ± 19.4
F8	0.75	0.4	12	36.0 ± 1.0	43.0 ± 0.6	7.6 ± 0.2	Pass	85.1 ± 3.1	59.0 ± 0.9	263.3 ± 14.8

All data represent mean ± SD (n = 3).

**Table 3:** Descriptive tables of demographic variables.

Group	Gender	Frequency	Percentage	Age (Mean ± SD)
Group 1	Male	9	60%	46.07 ± 10.6
	Female	6	40%	
Group 2	Male	8	53.3%	44.87 ± 7.1
	Female	7	46.7%	

**Table 4:** Inter-group comparisons between baseline and 14-day follow-up scores.

Parameter	Group	Baseline		Follow-Up		*p* Value ^‡^
		Mean	SD	Mean	SD	
Plaque score	Control	1.89	0.31	1.55	0.28	0.0001
	Test	1.67	0.49	1.15	0.23	
	*p* value ^†^	0.140				
Gingival index	Control	1.99	0.32	1.64	0.29	0.0003
	Test	1.70	0.45	1.25	0.23	
	*p* value	0.053				
Pocket depth	Control	5.53	0.52	4.40	0.63	0.0002
	Test	5.47	0.52	3.27	0.59	
	*p* value	0.483				

^†^ *p*-value for inter-group comparisons at baseline; ^‡^ *p*-value for inter-group comparisons at 14-day follow-up.

## Data Availability

Not applicable.
